# Managers as peer workers’ allies: A qualitative study of managers’ perceptions and actions to involve peer workers in Norwegian mental health and substance use services

**DOI:** 10.1186/s13033-023-00588-5

**Published:** 2023-06-12

**Authors:** Kristina Bakke Åkerblom, Torbjørn Mohn-Haugen, Rita Agdal, Ottar Ness

**Affiliations:** 1 Western Norway University of Applied Sciences, Bergen, Norway; 2 Erfaringssentrum, National Peer Worker Organization, Oslo, Norway; 3 Department of Education and Lifelong Learning, Norwegian University of Science and Technology, Trondheim, Norway

**Keywords:** Peer workers, Management, Mental health and substance use services, Service transformation, Boundary-spanning, Co-creation, Qualitative study

## Abstract

**Background:**

Citizens with experience and knowledge about what it is like to use mental health and substance use services are increasingly employed within similar services as peer workers. Peer workers are portrayed as achieving societal obligations and help ensure that the outputs from service provision are more effective. Even though peer workers have worked in mental health and substance use services for a while, few studies have focused on exploring managers’ experiences and perspectives about involving peer workers. This knowledge is needed because these managers can enable and hinder equitable involvement and collaboration with peer workers.

**Methods:**

A qualitative explorative study was chosen to explore the following research question: *How do managers in Norwegian mental health and substance use services experience, relate to, and embrace peer workers as assets in these services?* A researcher (Ph.D. student) and a coresearcher (peer worker) conducted four online focus groups with a strategic selection of 17 Norwegian mental health and substance use services managers who had some experience with the involvement of peer workers in their organizations.

**Results:**

The results identified using systematic text condensation are as follows: [1] *Peer workers boost the ongoing shift toward increased service user involvement.* [2] *Peer workers are highly valued in the service transformation process.* [3] *Managers involve peer workers as partners in co-creation.* The results show that managers connect with peer workers and facilitate their involvement in collaborative activities across the service cycle. Peer workers’ proximity to service users and bridging capacity is highlighted as the reasons for their involvement. Thus, peer workers are involved in co-defining challenges, co-designing potential solutions, co-delivering those service solutions, and, sometimes, co-assessing service solutions to rethink and improve services. As such, peer workers are considered partners in co-creation.

**Conclusion:**

As managers involve peer workers, they increasingly discover peer workers’ value, and because peer workers are involved, they increase their skills and capacity for collaboration. This research strengthens the knowledge base of the perceived value of peer workers’ roles, bringing in new perspectives from management about utilizing and evaluating peer worker roles.

## Background

Worldwide, citizens’ mental health needs are high, but current responses are insufficient and inadequate [1]. Individual and societal challenges resulting from mental health problems and substance use can be considered wicked or complex problems as they are intractable, unpredictable, have no single and simple solution, and, thus, can be challenging to address [2]. A suggested response when approaching such complex problems is collaborative practices involving the relevant and affected actors working together in creative problem-solving [3]. The relevant and affected actors are either affected by the situation or possess the appropriate knowledge and resources to contribute to a solution. In mental health and substance use services, one strategy to increase services’ responsiveness to service users’ needs and wants is employing citizens with lived experiences of similar challenges and service usage as peer workers [4]. Peer workers are characterized by the currently being or previously being affected by mental health challenges and have either overcome or learned to live well with them [5]. Thus, these individuals might possess relevant knowledge about potential solutions.

In mental health and substance use services, collaborative practices are well established as principles [6, 7]. Peer workers enter multidisciplinary organizations and often engage directly in interdisciplinary teams [8]. Peer workers’ involvement aligns with the new dominant direction in mental health service delivery, the recovery-oriented approach [9]. Recovery-oriented approaches highlight a partnership model involving peer workers, and their involvement is identified to increase the service user involvement [6]. Peer workers emphasize service user choice and autonomy and exercise voice, control, and influence over service delivery and development [10]. Peer workers are known to bring benefits and increase personal value to service users [11–14] and, as change agents, assist services in moving toward recovery-oriented service delivery [15, 16].

As employed within mental health or substance-use services, peer workers can act as the representatives of service users, who are likely to benefit from these services or their actions on behalf of the services [17]. Peer workers have a position ‘in between’ the service users and the services system. This intermediary position is perceived as one of the most significant reasons for their success [4] because they can bridge [18–22], link [8, 12, 23], and facilitate communication between the service users and the service system. By increasing service users’ access to resources within the service system [24], peer workers improve the services’ ability to tackle social needs [14, 24, 25]. Hence, peer workers’ representation can address service inequalities.

Even though peer workers are often depicted as having the power to drive social change, research has revealed a potential resistance to the integration of peer workers [9, 21, 26] and that peer workers’ ability to impact mental health service systems and delivery meaningfully is limited [9, 26–28]. Studies have begun identifying whether and how peer workers perform unique roles and functions [16]. Because peer worker involvement differs substantially across contexts, so does their potential to generate the inputs and affect service delivery and development [29].

In Scandinavian countries, peer worker involvement and practices are at an early stage [30–32]. In Norway, the context for the current study, there are still no national standards for the regulation, certification, or training of peer workers [30]. However, Norwegian policy aligns with policy found around the Western world [18] and has enshrined service user participation in the design and delivery of mental health and substance use services. Anyhow, Norwegian white papers do not describe peer workers’ roles or activities [33, 34]; this may give managers substantial room for interpretation and action.

The role of public managers in leading collaboration to achieve public value has received significant attention [35]. However, knowledge about how mental health and substance use services managers relate to and embrace peer workers’ knowledge and skills to benefit individuals and society is scarce. Few studies have focused on gaining information about managers’ perspectives [9, 36], experiences [36], or actions when it comes to involving peer workers [37]. One study suggests that the degree of management exposure to peer workers was essential for their understanding and commitment to applying them [38]. For this reason, more knowledge is needed about how managers who have gained experience with peer workers understand, commit to, and welcome them into collaborative practices in mental health and substance use services. The present research can offer promising perspectives regarding peer workers’ roles and involvement in collaborative practices in mental health and substance use services.

Collaborative practices in response to wicked or complex problems have received considerable attention in public sector innovation studies [39]. These approaches are denoted by the concepts of co-production and co-creation, which are often used interchangeably [40]. Yet, a split between these concepts can facilitate comparing peer workers’ involvement in collaboration practices in different contexts. In the present study, we use co-production to describe the collaboration involving peer workers and service users in service delivery [40]. In contrast, we use co-creation to refer to a broader involvement of peer workers in collaborative efforts, starting in the early phases of the service cycle, such as commissioning and design, combined with involvement in delivering those service solutions [39, 41, 42].

This split is further supported by a distinction Voorberg and colleagues (2015) used to describe various citizens’ roles in collaborative efforts as co-implementors, co-initiators, and co-designers. These researchers suggest using the term co-production for the involvement of citizens in the co-implementation of services and co-creation for the involvement of citizens as co-initiators or co-designers. Furthermore, they point out that citizens involved as co-implementors in the late stages of the service cycle will have less influence than citizens involved in the early stages as co-designers and co-initiators [40].

By and of itself, involvement in the late stages of a service cycle, like in service delivery or implementation, does not disrupt the common wisdom or established practice or lead to stepwise and innovative changes in a particular context [43]. On the contrary, co-creation efforts have an innovative dimension [44]. Following this, peer workers’ prospect of influence will be more significant in earlier phases of service development than in service production processes. This calls attention to peer workers’ involvement in the early stages of the service cycle as essential regarding their potential to impact the services they set out to change.

Thus, the current study explores managers’ perspectives on utilizing and evaluating peer workers’ roles in collaborative practices in mental health and substance use services. The specific research question is as follows: *How do managers in Norwegian mental health and substance use services experience, relate to, and embrace peer workers as assets in the services?*

## Methods

### Study design

A qualitative explorative study was chosen, specifically with a social constructionist stance [45]. In attempting to make sense of the social world, social constructionists view knowledge as constructed instead of created [45]. As such, the construction of understanding and meaning is created in encounters between people in social interactions, implying that knowledge production is not a neutral process but is shaped by positioning and power relations [45]. As the data collection method, we chose to use focus group interviews, which place the interaction between the participants at the center rather than the statements of individuals. The focus groups have proved helpful in identifying shared experiences and perceptions, including different perceptions [46]. We find this stance fruitful in the current study, which focuses on managers within mental health and substance use services’ understanding and attitudes toward peer workers’ involvement.

The focus groups were conducted on the online platform Zoom. This made it possible for the participants across significant geographical distances to participate in the same interviews. In two focus groups, some managers knew each other from earlier, while in the other two groups, all the managers were new to each other. In addition, there was a mix of experienced managers who had been early adopters of peer workers and new managers or managers with less experience in this respect. Online focus groups share the same principles as traditional focus groups, which means that social interactions between participants are essential. We noticed that the conversation between participants followed more turn-taking because they had to turn on and off their computer microphones; hence, this communication appeared less spontaneous than in a physical focus group. This may mean that group composition and dimensions such as power and hierarchy became less prominent as the participants waited for their turn to speak. Yet a weakness of conducting focus group interviews online is that the information that emerges only provides an indirect representation of selected aspects of what is going on between the participants.

### Participants and recruitment

The present study’s strategic selection aims to gather participants with substantial experimental knowledge of managing peer workers. This study means all participants have experience with the inclusion of peer workers. The participants were 17 managers from Norwegian mental health and substance use services. Emphasizing diversity [46], still not a pre-planned purposive sample, the participants ranged from being in a manager position in a year to be in a manager position for a decade or more. Furthermore, they were a mix of strategic managers (working within the services) and executive managers (working at the organizational level). There was also great diversity in age. Six of the participants were male, and eleven were female.

The participants were recruited via e-mail to organizations and distributed to stakeholders and managers. Stakeholders could be peer and nonpeer workers who forward information about the project to managers. The e-mail invitation explicitly stated an interest in learning from the experiences of managers who had experience recruiting peer workers and working with or had executive responsibilities for peer workers.

### Data collection

The focus group interviews were conducted a couple of days consecutively to a week apart in May and June 2021. The participants were divided into four groups with four to five managers. The discussions were facilitated by the current paper’s first and second authors, as informed by a semistructured interview guide they prepared together. The first author (Ph.D. student) has former experience as a manager within similar services, and the second author has experience as a peer worker (coresearcher). The collaboration between these authors started several years ago, related to a common interest in developing services in partnership with peer workers. Their common ground and preconceptions might be essential but not beneficial for all participating managers. While a common ground might have been valuable in facilitating good conversations, it also may have limited some participant comments.

Nevertheless, the facilitators’ shared understanding, yet different positions, backgrounds, and experiences were well communicated at the beginning of each group. The first and second authors’ impression was that this created a good atmosphere and opened communication in the focus groups. Still, we cannot rule out that it could imply that the participants responded in line with what they thought was the researchers’ expectations.

### Data analysis

All recorded focus group interviews (n = 4) were transcribed verbatim by a professional transcription service and reviewed for accuracy by the first author. The focus group interviews were imported into NVIVO 20 qualitative analysis software not to generate coding but to organize and quickly assess the study’s information, including transcripts and memos. The analysis followed systematic text condensation [47], a descriptive and explorative method following a four-step procedure for analysis. The first step, which all authors conducted, was to identify the preliminary themes that emerged spontaneously from the material. Taking these initial themes as a starting point in step 2, a meeting was arranged between the authors to study the data material more closely and organize it by analyzing statements for statements and categorizing them into groups of meaningful units. The first author identified meaning units in the original text, decontextualized them from their original context, sorted them by codes, and classified them, which resulted in the final themes. Subsequently, in step 3, the first author extracted the meaning units and rewrote them as continuous text in the first person for each theme (condensates). Finally, in step 4, the condensates were re-narrated in a third-person format and recontextualized to “elucidate the research question” [47]. As a result, an analytic text was prepared to present the main ideas within the material concerning the phenomenon in question. Then it has been illustrated by excerpts from the original interviews to represent the voices of participants. The results were validated against the original transcripts and reviewed and accepted by all the authors.

### Research ethics

The study was ethically approved by the Norwegian Centre for Research Data (Case No. 638,935). All managers participated voluntarily in the focus group through an informed consent process, which was a requirement for participation. They chose to answer an e-mail request from the first author or after being tipped off about the study by other managers or peer workers in their organization. They all replied to the first author directly and gave their written consent to participate. Participants were offered the opportunity to contact the first author after the interview. They have all been anonymized.

## Results

Using systematic text condensation, our analysis [47] identified three key categories describing how managers experience, relate to, and embrace peer workers as assets in the services [1]. *Peer workers boost the ongoing shift toward increased service user involvement*; [2] *peer workers are highly valued in the service transformation process*; and [3] *managers involve peer workers as partners in co-creation.* In addition, we identified distinct subthemes, which will be reflected in the subheadings linked to the key results. See Table 1 for an illustration of the categories and their related subthemes.

**Table 1:** Illustration of the results.

Themes	Subthemes
**Peer workers boost the ongoing shift toward increased service user involvement**	• Managers facilitate peer workers involvement • Benefits to the organization, nonpeer workers, and service delivery
**Peer workers are highly valued in the service transformation process**	• Peer workers? contextual knowledge is vital when redefining services • Peer workers facilitate communication and build bridges
**Managers involve peer workers as partners in co-creation**	• Managers commit to involving peer workers • Challenges when involving peer workers as partners in co-creation

### Peer workers boost a shift toward increased service user involvement

The managers clarified that the focus on service user involvement had increased significantly over the past few years. Furthermore, they stated how peer workers were essential to this shift in different ways. Managers described how they had instantaneously to gradually concluded that it was necessary to join in on what they described as an “*ongoing shift toward an increased focus on service user involvement*.” One way these managers approached this was by employing peer workers. Some managers upheld this shift toward the context that professionals in the services over time had been *“too poor at bringing in the service user voice and perspective”* and the need for more service user knowledge. One manager expressed, *“To solve the complex challenges ahead of us, we need more knowledge and different kinds of knowledge.”*

The managers described how they had already gone through a “journey” to where they are today. Some described how they, only a few years ago, perceived that employing peer workers could be risky to both peer workers’ and service users’ health and well-being.

#### Managers facilitate peer workers’ involvement

Several managers described how they, in different ways, facilitated peer workers’ involvement in the services and prepared both workplaces, nonpeer workers, and peer workers. Some managers said they had “*worked with the advantages and disadvantages of employing peer workers”* before employing them. Other managers explained that they had established dedicated nonpeer workers at the organizational level responsible for preparing and facilitating peer workers’ involvement across their organization.

Most managers also confirmed that they had strengthened the peer workers’ voices by focusing on training and supervision to enable peer workers to become more confident in their roles. Furthermore, several managers highlighted how they used peer workers at all levels of their organization; some also commented that they would like training for peer workers to pay more attention to various forms of involvement in the services besides functioning as service providers.

#### Benefits to the organization, nonpeer workers, and service delivery

Several managers discussed how peer workers’ entrance into the services led to lived experiences with mental health or substance use challenges no longer considered a risk or problem but a valuable resource. One manager elaborated on how they were not allowed to ask potential employees when interviewing about their background and experiences only a couple of years ago: *“Now, I can tell them that personal experiences with mental health or substance use challenges are something we value in our organization. And that it can be considered an advantage.”* Other managers said they had started to put it into all their announcements of nonpeer positions that personal experiences with mental health or substance use challenges could be a favored position. Another manager said, “*In our organization, personal experiences with mental health or substance use give some status.”* The managers also agreed that nonpeer workers with former experience with mental health or substance use challenges were viewed as more skilled and had considerable authority in their organizations. Perhaps because of this, some managers also reflected on how nonpeer workers started using their former experiences and background in their workplace and exposed the personal experiences they had earlier chosen to hide.

Furthermore, the managers called attention to how peer workers’ involvement humanized the services by challenging *how* services are provided, describing peer workers as a driving force in the transformation toward more inclusive and service user-oriented service delivery. Some managers discussed how the professional language used to be dominant in these services led to significant resistance and that undesirable language use had changed when peer workers entered the workplace. The managers further illuminated how peer workers also helped nonpeer workers understand that it is possible to meet citizens differently because they, as peer workers, approached persons and situations in slightly different ways. The managers describe how peer workers typically emphasized service user control and autonomy and communicated how service users could reduce the distance between themselves and the service system: “*It may be to use other words or methods to engage with our service users.”* In addition, they explained how peer workers often could function directly as advisors to nonpeer workers in different ways by sharing their knowledge and perspectives.

Furthermore, the managers stressed how they had gone through a journey where peer workers’ “voice and say” had become more vital as peer workers became a regular part of their workplaces. In different ways, the managers noted that peer workers soon became the ones who stopped, asked questions about practices, and challenged current practices. One manager said, “*Peer workers ask the essential questions not requested earlier.”* Another manager followed up on this: *“Or questions that may not have been asked frequently enough.”* The managers considered these questions the most fundamental, such as *“why do we do what we do”* or *“say as we say.”* The managers described how these questions could disrupt and lead to extensive dialogue in their services. However, most managers seemed to experience somehow that these dialogues prepared and enabled the services to resolve difficult situations with service users. In addition, because of peer workers’ questions, the managers described that nonpeer workers got the opportunity to reflect on their practices and see their ways of doing from a new perspective. However, some managers also explained how “*bold peer workers could be perceived as threatening to some nonpeer workers.*” A manager further stated that to counteract this, peer workers must function as a supplement to nonpeer workers. “*Peer workers should not take over the nursing task but close the gaps in our treatment offerings.”* This manager further stressed that such a position could lead to less resistance from professionals and perhaps help them explore how peer workers’ competence could complement their professional competence.

### Peer workers are highly valued in the service transformation process

Across the focus groups, the managers viewed peer workers’ role as central to service transformation. They presented the involvement of peer workers as a strategic investment, or a means to specific improvements. However, what particular value or contribution they were discussing was often unclear. This could also mean that it was opaque to the managers themselves, changing over time, or linked to the various activities peer workers performed. The managers frequently enhanced how peer workers increased cost-effectiveness, indicating their ability to boost service users’ say and involvement in decision-making. Others referred to peer workers as improving the quality of services. At the same time, some managers argued that peer workers’ involvement was legitimizing the services. Additonally, they told how their involvement in screening and generating ideas increased the likelihood of these ideas gaining acceptance by the service users. Most managers justified peer workers’ involvement by combining arguments based on seemingly different ideological approaches, such as consumerist or democratic [48].

Although most managers conveyed that they initially employed peer workers to work directly with service users, several described how they gradually involved peer workers in other activities and “at the managers’ table” when prioritizing, designing, and evaluating existing service offers: “*Involving peer workers has helped us ‘tune in’ our services to those we are there for and keep the spotlight on how to improve our services.”* The managers explained how peer workers put other issues on the agenda. One manager said, “*Earlier when developing new service offers, we constantly added what we had learned in our education. But these things are completely different from what peer workers are concerned with.”* Some managers further shared how because peer workers tune in and adopt the services to their citizens’ groups, they can engage with those citizens they could not reach in the past.

#### Peer workers’ contextual knowledge is vital when redefining services

The managers justified peer workers’ involvement with their context-based experiential knowledge that was claimed to be essential in the service delivery and redefining of the services in which they were employed. The peer workers were told to contribute knowledge and skills that enable services to adjust and “tune in” the overall service offered to the target group. In addition, they brought context information and abilities that assisted the services in approving existing services or designing the best new service solutions. Some managers commented that they had experienced that external user representatives from user organizations seldom brought in such knowledge, perhaps because their political mandate often seemed to control what they focused on in the collaboration.

The managers further discussed how peer workers’ contextual knowledge unfolds and that it is necessary for them when adjusting and developing the services. In different ways, the managers told they had seen how peer workers’ knowledge and skills had been evoked as they recognized specific situations or needs that their service users might have. In addition, several managers discussed that, for contextual knowledge to be utilized and valued in the services, peer workers’ proximity to the provided service was essential. As one manager said, “*We have benefited most when our peer workers have identical experiences as our target group.”* Some managers further declared that they saw it as a prerequisite for peer workers’ involvement and that they *“only will employ peers with experience of similar services as they offer.”*

Furthermore, some managers also said they looked for peer workers familiar with the specific geographic area in which they would work. A manager said, “*People who grew up in a place know what’s going on in that area*.” This was supplemented by the statement, *“They will know where to buy drugs or can identify persons and resources.”* The managers further discussed how peer workers within an area or district could better reach the target group and open up the dialogue with the citizens for the services meant.

#### Peer workers facilitate communication and build bridges

Most managers highlighted how peer workers facilitated communication and built bridges between service users and the service system. Some managers emphasized peer workers’ helpfulness and bridging function because of their local knowledge of a context or environment. Yet all managers seemed to agree that peer workers could reach out and get in touch with citizens for whom their various services were meant. One manager said, “*Peer workers are essential, especially for those service users lacking trust in the service system.”* These citizens were typically told to be persons who might have felt overwhelmed by a system or who, over time, had experienced not being listened to or not believed in.

### Managers acknowledge and involve peer workers as partners in co-creation

Several managers described how they involved peer workers in roles and activities across the phases of the service cycle—from initial problem definition, design, delivery, and assessment [49]—several highlighted peer workers’ vital function in service development. In different ways, the managers revealed they involved peer workers more broadly across the service cycle than as providers at the point of service delivery. Aligning with this, most of the managers in our focus groups revealed how they regularly included peer workers on various committees and collaborative groups at a higher level in their organization or collaborative groups working across services or sectors. This work was told to initiate and commission new, often combined, service offers or assess and adjust new offers to existing services. Several managers discussed how their organizations’ projects, organizational change, or service development processes no longer occurred without peer workers involved in significant positions. One manager stated, *“In our organization, we consider peer workers a fourth factor in developing services.”* This manager further explained that when they came together to explore new service solutions or negotiate and reallocate resources, they were obligated to bring their local stewards and safety representatives. In addition, they (managers) also chose to involve peer workers. Yet one of the managers also expressed concerns about what he described as “*deliberately letting peer workers replace representation from service user organizations.”*

In addition, some managers declare how they had developed their own service user boards in their organizations to get systematic inputs on service design and resource allocation. When the managers described who participated in these service user boards, it seemed to consist of a mix of existing service users and peer workers. Some managers further confirmed that they had handed over responsibility for leading those boards to their peer workers.

#### Managers commit to involving peer workers

The managers talked about how they involved peer workers in various activities and how their continuous interactions enabled trustful and robust relationships. Those managers who participated in our focus groups said they had worked closely with peer workers from their entrance and still did because they perceived that peer workers’ perspectives had become a necessary corrective for them in their practice as managers. In different ways, the managers explained how they established and nurtured those close connections because it would increase the likelihood of peer workers daring to *“see the services in the cards.”* Most managers embraced how they needed peer workers who could take on a “*critical position.”* Some also highlighted how they viewed this as a crucial part of the peer workers’ role.

However, other managers discussed how peer workers who challenged the services’ ways of ‘doing and thinking’ could also increase the trust between the service users and the service system. Through this, those peer workers could bridge gaps with service users and improve the services’ general credibility. Still, some managers brought to the discussion that they had experienced that peer workers’ questioning of existing practices also could reinforce nonpeer workers’ feeling threatened by them.

The managers were concerned with reducing what some referred to as “the traditional power imbalance” in the services. One manager explained how he deliberately employed peer workers before a psychiatrist: “*Peer workers should not feel they must step on their toes to be part of the professional community.*” This was followed by a discussion between the managers about how successful collaboration depended on mutual understanding and respect. In different ways, the managers explained how they tried to equalize peer workers’ positions with their nonpeer colleagues to facilitate meaningful collaboration and create a common ground for equal-footed collaboration.

Most of the managers talked about how they, as managers, took up a special responsibility to encourage peer workers’ involvement and paid attention to demonstrating their trust in peer workers. Some suggested this as an act that would strengthen peer workers’ overall positions in the service systems. Other managers also stated that they “*from time to time had to reassure peer workers that their service user perspective was essential.”* The managers in our focus groups seemed to commit to involving peer workers in meaningful ways. Additionally, the managers discussed how they chose to involve peer workers because these individuals are closer to their services than traditional user representatives from user organizations. At the same time, some managers emphasized that those user representatives were often involved in addition to their peer workers.

#### Challenges when involving peer workers as partners in co-creation

Some of the managers conveyed that involving peer workers was challenging and could be time-consuming for them as managers, especially at the beginning. The managers seemed to agree that, after some time, their efforts to involve peer workers in meaningful collaboration would be overshadowed by the benefits of involving them.

Furthermore, some managers discussed how a tradition of risk aversion was gradually replaced and that they, as managers, were increasingly encouraged to take more risks and explore collaborative efforts to solve challenging issues. As one manager expressed: *“We are still testing out how to utilize peer workers and see no end to using their expertise.”* As part of this discussion, some managers confessed that giving so much responsibility to citizens who recently had significant mental health and substance use challenges was initially a little scary. They also reflected that, only a few years ago, they all perceived that employing peer workers was too risky for both peer workers’ and service users’ health and well-being.

Yet other managers talked about how skewed power relationships between peer workers and nonpeer workers in the mental health service system made peer workers’ involvement in collaborative efforts demanding. One manager said, *“It is difficult to involve someone less educated to co-create with well-educated people on an equal ground and from the beginning.”* This statement was followed by a discussion between some managers confessing how easy it was to fall back on both using and valuing professionals’ competence the most.

Additionally, several managers discussed a connection between peer workers’ status in policy documents and their status in the services, highlighting the need for improved policy documents and how this would have helped them use peer workers. As one manager said, “*When it is a clear expectation how to understand and utilize peer workers in policy, the manager’s task is to make sure that it happens*.”

## Discussion

This study contributes to the current understanding of peer workers’ value for mental health and substance use services. Furthermore, it brings in new perspectives from managers who are experienced with peer worker inclusion on how to utilize peer workers’ roles in the services. In line with former research, Norwegian managers depict peer workers as increasing service user involvement [12, 50–52] and boosting the shift toward recovery-oriented services [9, 21, 50, 53, 54]. However, in the current study, the managers focus on the collaborative processes in which peer workers are involved and facilitate and expand their scope of involvement across the service cycle. Based on our findings, we discuss how managers prioritize the quality of collaborative practices to increase peer workers’ ability to impact service systems and how this may stimulate innovation.

### Peer workers’ role in the transition toward recovery-oriented services

Norwegian mental health and substance use managers described how these services fundamentally have changed in just a few years. They situated peer workers in a vital position in transforming toward recovery-oriented services. Managers in this study confirm earlier research about peer workers’ role in this shift [15, 16]. In essence, these managers depicted peer workers as a strategy to address service inequities [22] and compensate for the earlier unsatisfying interaction between the service users and the service system [55]. Managers in this study considered peer workers as representatives for their services present service user group [55–57]. As such, managers said, they employed peer workers who shared backgrounds or came from similar social contexts as their present service user groups. This seems to build on the assumption that the more identical peer workers’ experiences are to present service user groups, the more likely they will bridge the gaps [56] to those groups and increase their access to services [22, 55].

Equivalent to how peer workers’ similarities in backgrounds and experience were considered of immediate relevance when linking and bridging to service users, managers told how peer workers’ backgrounds as service users [5] were vital for their nonpeer colleagues because they learned to approach persons and situations in slightly different ways.

### Experienced managers expand the scope of peer workers’ involvement

While the international research literature often describes peer workers’ positions and functions as primarily focused on service provision [58–60], managers in this study depicted peer workers’ involvement in service development processes across the service cycle and at the strategic level essential. These managers described peer workers as engaged in shaping and commissioning services and implementing and delivering those services, which aligns with co-creation [39, 41, 42]. Moreover, some managers in this study told how they explicitly employed peer workers to engage at all levels in their organizations, serving in dual roles: as board and committee members and as service providers. Yet, other managers described how they gradually increased the scope of peer workers’ involvement across the service cycle. The managers seem to agree that peer workers’ valuable insights into service users’ needs [24] also assist the services in designing the best solutions [61, 62] and reasoned about peer workers’ contextual skills and insights into service users’ needs. This reasoning aligns with peer workers being “lead users,” described by Von Hippel (1986), as persons who can provide valuable insights into service users’ needs and “prototype” solutions for novel services [63]. Similarly, managers said that peer workers brought in knowledge and perspectives that helped them prioritize efforts differently, developing and transforming services to better meet the needs of their service user group.

### Managers prioritize relationship-building and continuous support

In the current study, managers focus on relationship building and continuous support with peer workers. Earlier research has stated that managers’ perceptions of peer workers’ benefits are essential when calculating whether to involve them in collaboration [36]. In this study, managers furthermore demonstrate their trust in peer workers [8] and facilitate their involvement in the collaborative processes. Moreover, former research has revealed a connection between whether ongoing support is prioritized and the perceived benefits of involving peer workers [18], which supports the action of managers in this study.

Assumingly, the managers’ attention and commitment to involving and supporting peer workers in collaborative interaction will likely improve the quality of the collaborative processes. Moreover, managers’ ongoing support and dedication can be necessary for peer workers to become a regular part of the service and establish long-term relationships with nonpeer colleagues [57]. Managers’ effort is furthermore likely to increase the peer workers’ trust in the service systems, which is vital in collaboration [64]. Additionally, peer workers’ confidence and trust can be transferred to their service user group [56, 57].

### Peer workers’ ability to impact service systems

In the current study, the managers demonstrate trust in peer workers [8] and involve them as partners in co-creation, increasing their ability to impact the service systems. The broad involvement of peer workers at all levels in their organizations, described by managers, aligns with public sector innovation research suggesting service user involvement occurs at all phases of a (public) service lifecycle [24].

Moreover, managers describe how peer workers have a mix of tasks and activities and serve as members of boards and committees and service providers in dual roles. Peer workers moving back and forth between their workplaces and these boards or committees is likely to be productive at the point of service delivery and may, in addition, foster broader system change because peer workers can ensure their concerns are taken forward across the organizational hierarchy and considered within decision-making processes [67, 68]. Peer workers doing cross-boundary work align with boundary spanners in the public management literature [65]. Individuals who serve as boundary spanners in co-creation processes are considered essential [66]. The use and benefits of persons in such positions are believed to be enabled when engaging in various collaboration platforms [66]. Following this argument, peer workers serving in dual roles as members of boards and committees and service providers can be vital for their ability to impact service systems.

#### Peer workers as co-creation partners disrupt the existing practices

Involving peer workers in ways that challenge or disrupt the established practice in mental health and substance use services will need more than continuous support from their managers. First, when peer workers are involved as partners in co-creation, this is likely to have an adverse outcome for some actors. Thus, when peer workers increase their ability to impact, in the same way, other actors can lose control of tasks, activities, or their previous roles. Several studies have pointed to a power imbalance between peer workers and nonpeer workers or professional actors in mental health and substance use services [60] and how peer workers are not considered equal-footed partners. As such, peer workers’ involvement will presumably also need support from other actors, like their nonpeer colleagues. Besides, their involvement will need permission from the policy [70].

On the contrary, we could imagine that the continuous support from managers will lead peer workers to be connected, yet also become more loyal to covering up inadequacies in the services than pointing out errors and shortcomings. Several studies have problematized peer workers’ risk of being co-opted by their employing mental health and substance use organizations [54, 70, 71]. Likewise, how peer workers’ intermediary position between service users and nonpeer colleagues means they risk becoming more like their nonpeer colleagues [32] than the service users they were intended to represent.

Over and above, peer workers’ roles and involvement may challenge service users’ participation through user organizations. Peer workers’ involvement is less described in the Norwegian policy documents [33, 34], while user organizations bringing in the service user perspective at a system level still is the traditional way in these services. This kind of involvement of user representatives happens by involving them in committees at the system level. In these committees mental health and substance user service organizations inform them so that they can voice their opinions and object to ideas and proposals put forward by the service organizations. As these persons represent their user organization’s view, they risk, to a lesser degree, becoming co-opted by the service organizations.

Yet, in the current study, managers said they preferred peer workers because they were considered in a position of more relevant knowledge and were easier to collaborate with. When peer workers are employed within the services, they learn to see the service systems from the inside, gain organizational skills, and establish relationships with nonpeer workers, managers, and other stakeholders. Moreover, peer workers’ position enables them to engage in dialogue-based co-creation of results over a more extended period, and this collaboration is entirely different from voicing their opinions and objecting to ideas and proposals put forward or not by managers.

Besides, the extensive focus on implementation issues and barriers in the research literature describing peer workers [29] adds to the idea that peer workers’ involvement is primarily considered a virtue, which does not need to be legitimized by referring to external objectives. The collaborative efforts involving peer workers have a normative appeal [40] because the involvement of peer workers as ‘relevant and affected’ actors is essential for democratic purposes [40]. In this study, managers highlight the benefits peer workers bring – yet they did not pay considerable attention to the potential disvalue peer workers’ might entail, nor the challenges of their involvement. This aligns with a trend in the literature on (public) value creation, which primarily focuses on the positives, assuming value to be created [64]. However, suppose managers pay more effort to employing peer workers than exploring how to utilize their competence in the most meaningful way and evaluate their outcomes. In that case, the symbolic function might be high, while the effect can be low.

### Limitations

The current study is limited to one country, Norway. Because peer workers employed in services are still in an early phase, this may also mean that our selection of managers typically consists of the most dedicated who have started early, which may picture a practice more unique than expected. Because the method for collecting data was focus group interviews, we cannot rule out that the managers paid great attention to positioning themselves, exaggerating what they considered positive in their practice to impose on other participants. Hence, the managers might have presented their intentions more than their actions. Furthermore, as with most qualitative research, the current study has a relatively small number of participants.

### Direction for future research

While the qualitative data collected from managers’ perspectives can contribute to theory and practice, this could be supplemented with quantitative data about the actions of managers responsible for implementing peer workers. Furthermore, it would be helpful to gain more in-depth knowledge about these collaborative practices from the nonpeer workers’ and peer workers’ perspectives, especially those who also serve or have experience as traditional user representatives through user organizations. Primarily peer workers’ impact has been documented through interviews study, and there is generally little quantitative research about how peer workers impact various influential factors. As our understanding suggests that peer workers’ various roles and involvement have a different impact, more knowledge about designing and evaluating effective peer workers’ roles is needed. Then, building on this knowledge, it would be interesting to measure how strong the collaborative partnerships with peer workers are and if these partnerships can create service offers or new service solutions that are called for by the service users, - and have the desired effect.

### Concluding remarks

The findings from the current study show that managers in Norwegian mental health and substance use services benefit from peer workers in shifting toward recovery-oriented services. The managers focus on the quality of the collaborative processes in which peer workers are involved and on facilitating and expanding their scope of involvement across the service cycle. Managers’ attention to improving the quality of the collaborative processes and commitment to involving peer workers in close and deep collaborative interaction can increase the likelihood of conflicts being constructively managed and the exchange of resources and ideas that will produce clear and tangible results. Furthermore, as employed within the service system, peer workers develop new skills and expand their knowledge of the mental health system. Even though peer workers undoubtedly risk being co-opted by their organizations or gradually become more like their non-peer colleagues, managers embrace peer workers’ position as representatives, in-between the service user group and their nonpeer colleagues, as reasons for involving them as partners in co-creative practices. Suppose peer workers are engaged in the collaborative processes as broad, deep, and close as managers describe. Besides challenges that may be reflected in the actual reality of co-creative practices that need to be dealt with, peer workers’ involvement as partners in such practices will be a more potent driver of innovation than traditional service user participation through user organizations. Peer workers as partners in co-creative practices might have great innovative potential and move beyond tokenistic participation [68], in line with the intention of the recovery approach [69].

## Data Availability

All data will be available upon publication.
